# Case Report: transjugular intrahepatic portosystemic shunt combined with hemodialysis for refractory ascites treatment in a patient with idiopathic non-cirrhotic portal hypertension and uremia

**DOI:** 10.3389/fmed.2025.1607521

**Published:** 2025-07-18

**Authors:** Ying Li, Xin Quan, Hao Wu

**Affiliations:** Department of Gastroenterology and Hepatology, West China Hospital, Sichuan University, Chengdu, Sichuan, China

**Keywords:** transjugular intrahepatic portosystemic shunt, refractory ascites, idiopathic non-cirrhotic portal hypertension, uremia, hemodialysis, case report

## Abstract

Transjugular intrahepatic portosystemic shunt is a standard treatment for refractory ascites (RA) in patients with cirrhosis. Idiopathic non-cirrhotic portal hypertension (INPH) is a disorder of unknown etiology, clinically characterized by features of portal hypertension. The current therapy is limited to managing portal hypertension and is recommended to be referred to as cirrhosis. Given the elevated risk of overt hepatic encephalopathy (OHE) post-TIPS, TIPS placement is limited in cirrhotic patients with concurrent acute or chronic kidney disease. However, patients with INPH exhibit better liver function and ammonia metabolism than those with liver cirrhosis. The efficacy of TIPS for RA in INCPH patients with uremia on dialysis remains uncertain. We present a case of TIPS placement for RA in a patient with INPH on maintenance hemodialysis for uremia, aiming to explore therapeutic advancements and enhance quality of life in this challenging population.

## Introduction

Idiopathic non-cirrhotic portal hypertension (INCPH) is a disorder characterized by features of portal hypertension (1). It originates from various histopathological conditions that have been termed hepatoportal sclerosis, non-cirrhotic portal fibrosis, nodular regenerative hyperplasia, or incomplete septal fibrosis/cirrhosis (2). It has been associated with the use of immunosuppressive drugs for the management of autoimmune and hematological disorders, as well as the increased prevalence of treated HIV infection (3). Compared to variceal bleeding, refractory ascties (RA) is less common in patients with INPH, which may be related to normal serum albumin in these patients (4). Given the current paucity of research data on INCPH, clinical management based on cirrhosis guidelines is recommended (5).

Transjugular intrahepatic portosystemic shunt (TIPS) is a standard treatment for portal hypertension-related RA in patients with cirrhosis (6). Its effectiveness in cirrhotic patients with uremia undergoing renal replacement therapy remains unclear due to most of randomized controlled trials about TIPS typically exclude these individuals (7, 8). The decision to place TIPS in patients with kidney dysfunction is recommended to base on a case-by-case basis and taking into account other predictors of outcome (9, 10). Studies indicated that renal dysfunction exacerbates the risk of post-TIPS overt hepatic encephalopathy (OHE) and is the most common predictive factor of an unfavorable response to TIPS in cirrhotic patients. However, patients with INPH exhibit better liver function and ammonia metabolism than those with liver cirrhosis. Furthermore, a comparative study of TIPS outcomes between INCPH and cirrhotic patients demonstrated that the INCPH cohort exhibited significantly lower rates of OHE and reduced mortality (11). Therefore, the efficacy of TIPS for RA in patients with INPH and renal insufficiency requires further investigation (12).

Hemodialysis is one of the most prevalent forms of renal replacement therapy in uremic patients. The initial intervention involves extending the duration or increasing the frequency of hemodialysis when RA occurs in patients with INPH and uremia (13). While hypotension may hinder its long-term implementation (14). Moreover, INPH is the primary cause and increasing ultrafiltration may not effectively alleviate RA.

RA significantly reduces patients’ quality of life and prognosis. The efficacy of TIPS combined with hemodialysis for RA in INPH patients with uremia remains unclear. Therefore, we firstly reported the case where TIPS combined with hemodialysis to alleviate RA in patients with INPH and uremia, aiming to explore therapeutic advancements and enhance quality of life in this challenging population.

## Case description

A 77-years-old female patient was admitted to our hospital complaining of recurrent abdominal distension for 3 years. Three years ago, the patient sought an abdominal ultrasound due to bloating, revealing minimal ascitic fluid and splenomegaly. Two months prior, worsening bloating and increased abdominal circumference led to intermittent paracentesis, yet bloating persisted. For further diagnosis and treatment, she was transferred to our hospital. The patient was diagnosed with chronic kidney disease (CKD) 8 years ago, which progressed to uremia 6 years ago, and she has since been undergoing regular hemodialysis 3 times per week by tubular arteriovenous fistula. She had a medical history of thalassemia. No other special family history was found. A physical examination revealed abdominal distension with positive shifting dullness and no tenderness.

**FIGURE 1:**
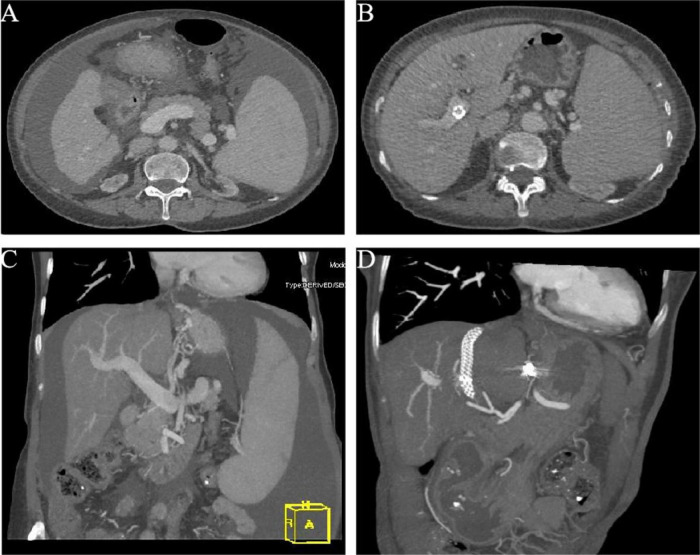
Abdominal enhanced computed tomography before TIPS **(A)** and after TIPS **(B)** in the case. Three-dimensional computed tomography (CT) images before TIPS **(C)** and after TIPS **(D)** demonstrate significant resolution of ascites post-procedure. TIPS, transjugular intrahepatic portosystemic shunt.

**FIGURE 2:**
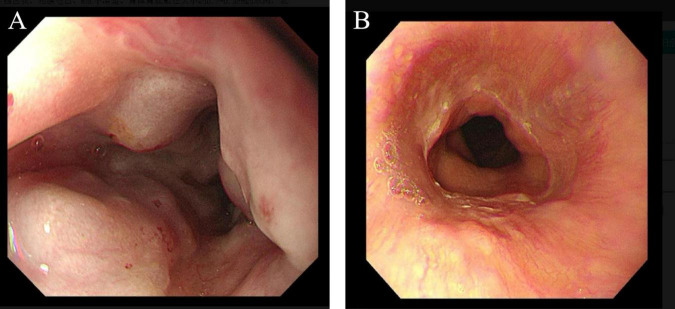
**(A)** Upper gastrointestinal endoscopy before transjugular intrahepatic portosystemic shunt (TIPS); **(B)** upper gastrointestinal endoscopy was performed at 6 months after TIPS placement.

Laboratory examinations were as follows: hemoglobin (HB), 6.5 g/dL; leukocyte count, 2.9 × 10^3^/μL; platelet count (PLT), 45 × 10^3^/μL; alanine aminotransferase (ALT), 5 U/L; aspartate transaminase (AST), 22 U/L; total bilirubin (TB), 12.6 μmol/L; direct bilirubin (DB), 4.4 μmol/L; albumin (ALB), 42.5 g/L; and creatinine (Cr), 301 μmol/L; glomerular filtration rate (GFR), 12.38 ml/min/1.73 m^2^; international normalized ratio (INR), 1.06; prothrombin time (PT), 11.9 s; and blood ammonia, 24.9 μmol/L. All viral hepatitis markers were negative. Laboratory evaluation demonstrated low-titer ANA positivity (1:100) with mixed speckled/homogeneous pattern, hypocomplementemia (C3 0.43 g/L, C4 0.13 g/L), and dysproteinemia (albumin 43.2%, γ-globulin 37.8%), with negativity for ANCA, ENA panel, anti-dsDNA, IgG4, serum immunoglobulins (IgG/A/M), ceruloplasmin, immunofixation electrophoresis, and autoantibodies associated with autoimmune liver diseases (anti-M2, SLA, LC-1, LKM, Sp100, gp210). The abdominal puncture fluid was clear and bright, and the serum ascites albumin gradient (SSAG) was > 11 g/L. Abdominal computed tomography (CT) suggested a substantial amount of ascites and severe bilateral renal atrophy (Figures 1A, C). Upper gastrointestinal endoscopy indicated severe esophageal varices (Figure 2A). Preoperative echocardiography revealed mild left atrial enlargement, slight dilation of the ascending aorta, and normal left ventricular systolic function (ejection fraction = 60%).

**FIGURE 3:**
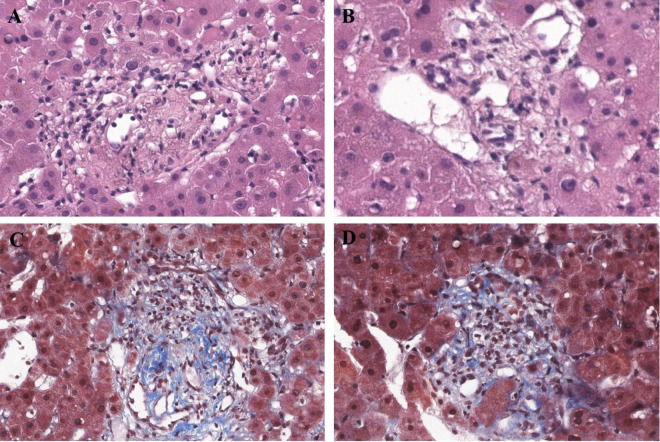
Histopathological features of obliterative portal venopathy/portal vein stenosis. **(A,B)** Hematoxylin and eosin staining, 40×; **(C,D)** masson trichrome staining (40×) demonstrating.

The frequency of hemodialysis was increased to five times per week and large volume paracentesis was performed, ascites occurred repeatedly. To further investigate the etiology and therapeutic approach for RA, we performed a liver biopsy and portal pressure gradient (PPG) measurement following paracentesis with albumin infusion. The PPG was 18 mmHg and liver biopsy revealed portal fibrosis (Metavir F1) in 10 portal tracts without bridging fibrosis, ductular reaction (CK7-negative), or significant inflammation. Vascular changes included interlobular vein dilation. Special stains excluded copper/iron/α1-antitrypsin accumulation, and immunohistochemistry was negative for HBV infection (HBsAg/HBcAg-negative) and plasma cell infiltration (CD38-negative) (Figure 3). TIPS placement was considered to reduce PPG and alleviate RA. An 8-mm viatorr-covered stent was deployed and PPG was reduced to 8 mmHg.

**FIGURE 4:**
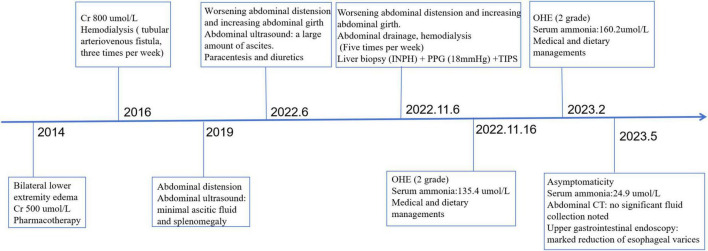
The patient’s diagnostic and therapeutic timeline.

During the 6-months post-TIPS follow-up, the patient demonstrated significant improvement in abdominal distension, appetite, and nutritional status compared to pre-procedure baseline. However, she experienced two episodes of grade 2 OHE, both precipitated by constipation. These episodes were successfully managed with medical therapy (lactulose and rifaximin) and dietary modifications. Follow-up abdominal CT at 6 months post-TIPS demonstrated significant resolution of ascites (Figures 1B, D). Upper gastrointestinal endoscopy performed concurrently revealed marked reduction of esophageal varices (Figure 2B). The diagnostic and therapeutic timeline is summarized in Figure 4.

## Discussion

Ascites is one of the clinical manifestations in patients with INPH, indicating an unfavorable prognosis (15). Ascites formation is primarily driven by portal hypertension and subsequent activation of the renin-angiotensin-aldosterone system (RAAS) (16). TIPS is effective in the management of RA by reducing the PPG, and the Baveno VII conference recommends that TIPS placement should be considered for patients with RA irrespective of the presence or absence of varices or a history of variceal hemorrhage (5).

Uremia refers to various signs and symptoms associated with generalized organ dysfunction caused by the accumulation of hazardous metabolites in the plasma (17). Significant oliguria and tissue edema represent cardinal clinical manifestations of uremia. Hemodialysis is one of the most prevalent forms of renal replacement therapy and significantly increases the life expectancy of patients (18). However, for patients with concomitant INPH, as in the case we reported, ascites persisted despite increasing the frequency of hemodialysis. Ascites formation involves multiple mechanisms: INPH increases intrahepatic vascular resistance, leading to passive transudation of fluid into the peritoneal cavity (4). Concurrently, elevated nitric oxide (NO) production activates both the RAAS and sympathetic nervous system, resulting in sodium and water retention (11). Uremia impairs renal excretory function, which exacerbates interstitial fluid accumulation, while retained uremic toxins further increase vascular permeability (19). TIPS reduces portal hypertension by creating a shunt between the hepatic and portal veins, thereby decreasing splanchnic congestion and lymphatic fluid production. This improves effective circulating volume and suppresses RAAS overactivation, ultimately mitigating ascites formation. Additionally, TIPS enhances the gradient for intraperitoneal fluid reabsorption into the vasculature. When combined with hemodialysis, it facilitates the removal of excess fluid and reduces venous pressure, further limiting ascites accumulation. Hemodialysis also clears uremic toxins, lowering vascular permeability and preventing additional fluid extravasation into the peritoneal cavity.

In cirrhotic patients undergoing TIPS placement, a preprocedural GFR below 90 mL/min is associated with increased post-TIPS mortality (12). Bissonnette et al. (20) also found serum creatinine, ascites as indication for TIPS were associated with death in cirrhosis. However, compared to patients with liver cirrhosis, patients with INPH may have better liver function and the ability to metabolize blood ammonia. Guidelines also recommended the management in INPH patients should be individual. Consistent with previous studies, OHE is the most common complication after TIPS. The patient we reported developed two episodes of OHE due to constipation, yet it could be acutely improved through diet and drug managements. In previous reports about cirrhotic patients combined with uremia, recurrent HE occurred after TIPS and were unresponsive to drug therapy (19). This may be related to the diameter of the stent, inadequate hemodialysis following TIPS, and impaired liver function. Hemodialysis may improve OHE in patients following TIPS via the removal of protein or non-protein-bound toxins (21). The patient immediately underwent enough hemodialysis after TIPS and the frequency was consistent with the preoperative frequency. The liver plays a pivotal role in the metabolism of serum ammonia and pre-TIPS deterioration of liver function also be a contributory factor to the recurrence of HE following TIPS. Compared with these cirrhotic patients, our patient had a preserved liver function. Additionally, a recent observational study by Nardelli et al. revealed that post-TIPS OHE did not increase patient mortality after the procedure (22). Therefore, TIPS placement can be considered to relief RA patients with INPH and uremia.

In conclusion, although RA in patients with uremia and INPH is complex and less common in clinical practice, TIPS should be comprehensively considered from the perspectives of improving quality of life and prolonging survival time. Our study provides a potential treatment option for such patients, and further clinical studies are needed to validate this approach.

## Data Availability

The raw data supporting the conclusions of this article will be made available by the authors, without undue reservation.

## References

[1] Khanna R Sarin S. Non-cirrhotic portal hypertension - Diagnosis and management. *J Hepatol.* (2014) 60:421–41. 10.1016/j.jhep.2013.08.013 23978714

[2] Guido M Alves V Balabaud C Bathal P Bioulac-Sage P Colombari R Histology of portal vascular changes associated with idiopathic non-cirrhotic portal hypertension: Nomenclature and definition. *Histopathology.* (2019) 74:219–26. 10.1111/his.13738 30129657

[3] Chang P Miquel R Blanco J Laguno M Bruguera M Abraldes J Idiopathic portal hypertension in patients with HIV infection treated with highly active antiretroviral therapy. *Am J Gastroenterol.* (2009) 104:1707–14. 10.1038/ajg.2009.165 19471257

[4] De Gottardi A Sempoux C Berzigotti A. Porto-sinusoidal vascular disorder. *J Hepatol.* (2022) 77:1124–35. 10.1016/j.jhep.2022.05.033 35690264

[5] de Franchis R Bosch J Garcia-Tsao G Reiberger T Ripoll C. Baveno VII - Renewing consensus in portal hypertension. *J Hepatol.* (2022) 76:959–74. 10.1016/j.jhep.2021.12.022 35120736 PMC11090185

[6] European Association for the Study of the Liver. EASL Clinical Practice Guidelines for the management of patients with decompensated cirrhosis. *J Hepatol.* (2018) 69:406–60. 10.1016/j.jhep.2018.03.024 29653741

[7] García-Pagán J Caca K Bureau C Laleman W Appenrodt B Luca A Early use of TIPS in patients with cirrhosis and variceal bleeding. *N Engl J Med.* (2010) 362:2370–9. 10.1056/NEJMoa0910102 20573925

[8] Lv Y Zuo L Zhu X Zhao J Xue H Jiang Z Identifying optimal candidates for early TIPS among patients with cirrhosis and acute variceal bleeding: A multicentre observational study. *Gut.* (2019) 68:1297–310. 10.1136/gutjnl-2018-317057 30415233

[9] Deltenre P Zanetto A Saltini D Moreno C Schepis F. The role of transjugular intrahepatic portosystemic shunt in patients with cirrhosis and ascites: Recent evolution and open questions. *Hepatology.* (2023) 77:640–58. 10.1002/hep.32596 35665949

[10] European Association for the Study of the Liver. EASL clinical practice guidelines on TIPS. *J Hepatol.* (2025) 83:177–210. 10.1016/j.jhep.2025.01.029 40180845

[11] Lv Y Li K He C Luo B Zhang B Liu H TIPSS for variceal bleeding in patients with idiopathic non-cirrhotic portal hypertension: Comparison with patients who have cirrhosis. *Aliment Pharmacol Ther.* (2019) 49:926–39. 10.1111/apt.15186 30820990

[12] Hamel B Guillaud O Roman S Vallin M Pilleul F Valette P Prognostic factors in patients with refractory ascites treated by transjugular intrahepatic porto-systemic shunt: From the liver to the kidney. *Dig Liver Dis.* (2014) 46:1001–7. 10.1016/j.dld.2014.06.013 25096966

[13] Gunal A Karaca I Celiker H Ilkay E Duman S. Strict volume control in the treatment of nephrogenic ascites. *Nephrol Dial Transplant.* (2002) 17:1248–51. 10.1093/ndt/17.7.1248 12105248

[14] Glück Z Nolph K. Ascites associated with end-stage renal disease. *Am J Kidney Dis.* (1987) 10:9–18. 10.1016/s0272-6386(87)80004-5 3300289

[15] Schouten J Nevens F Hansen B Laleman W van den Born M Komuta M Idiopathic noncirrhotic portal hypertension is associated with poor survival: Results of a long-term cohort study. *Aliment Pharmacol Ther.* (2012) 35:1424–33. 10.1111/j.1365-2036.2012.05112.x 22536808

[16] Bosch J García-Pagán J. Complications of cirrhosis. I. portal hypertension. *J Hepatol.* (2000) 32:141–56. 10.1016/s0168-8278(00)80422-5 10728801

[17] Nigam S Bush K. Uraemic syndrome of chronic kidney disease: Altered remote sensing and signalling. *Nat Rev Nephrol.* (2019) 15:301–16. 10.1038/s41581-019-0111-1 30728454 PMC6619437

[18] Planas R Montoliu S Ballesté B Rivera M Miquel M Masnou H Natural history of patients hospitalized for management of cirrhotic ascites. *Clin Gastroenterol Hepatol.* (2006) 4:1385–94. 10.1016/j.cgh.2006.08.007 17081806

[19] Michl P Gülberg V Bilzer M Waggershauser T Reiser M Gerbes A. Transjugular intrahepatic portosystemic shunt for cirrhosis and ascites: Effects in patients with organic or functional renal failure. *Scand J Gastroenterol.* (2000) 35:654–8. 10.1080/003655200750023642 10912668

[20] Bissonnette J Garcia-Pagán J Albillos A Turon F Ferreira C Tellez L Role of the transjugular intrahepatic portosystemic shunt in the management of severe complications of portal hypertension in idiopathic noncirrhotic portal hypertension. *Hepatology.* (2016) 64:224–31. 10.1002/hep.28547 26990687

[21] Hassanein T Tofteng F Brown R McGuire B Lynch P Mehta R Randomized controlled study of extracorporeal albumin dialysis for hepatic encephalopathy in advanced cirrhosis. *Hepatology.* (2007) 46:1853–62. 10.1002/hep.21930 17975845

[22] Nardelli S Riggio O Marra F Gioia S Saltini D Bellafante D Episodic overt hepatic encephalopathy after transjugular intrahepatic portosystemic shunt does not increase mortality in patients with cirrhosis. *J Hepatol.* (2024) 80:596–602. 10.1016/j.jhep.2023.11.033 38097113

